# Supplementary material for: Human ventromedial prefrontal cortex is necessary for prosocial motivation
Source: Nat Hum Behav. 2024 May 27;8(7):1403–16. doi: 10.1038/s41562-024-01899-4 (PMC11272586; doi:10.1038/s41562-024-01899-4)
Supplement: Supplementary file 1 — Supplementary Figs. 1–7, Tables 1–7 and Methods. [file 41562_2024_1899_MOESM1_ESM.pdf]

nature human behaviour

Supplementary information https://doi.org/10.1038/s41562-024-01899-4

Human ventromedial prefrontal cortex is necessary for prosocial motivation

In the format provided by the authors and unedited

Supplementary Methods

Statistical analysis

For the trial-by-trial measures, choices and force, maximal models contained fixed effects of recipient, group, effort, and reward, as well as all possible interactions. The maximal random-effects structure included all variables except group, and all interactions, in addition to a subject-level random intercept. Effort and reward were z-scored before being entered into the models and for the choice analysis only, effort was first squared, in line with the best-fitting computational model. As the maximal model was always too complex to converge, we used the buildmer package[1] (v2.3) to reduce models to the best-fitting terms, based on the default, likelihood ratio test criteria. The binary choices to work or rest were modelled with a binomial generalised LMM (GLMM) and we modelled continuous force exerted and success rates with LMMs. Participants' force was normalised as a proportion of their maximum to account for between-subject variability in force exerted and calculated as the area under the curve for the 3 second window in which they exerted force. Success rates were the average number of times participants successfully exerted the required force to gain the reward on trials they chose to work. Models of credits earned, the computational parameters (K and β), and success rates had fixed effects of recipient (self or other) and group (vmPFC, HC or LC), and a subject-level random intercept, as there is only one datapoint per participant per recipient. The GLMMs of credits earned and K parameters used a gamma distribution with log link function, to account for the nature of the data without transforming the raw values. In all models, group was coded using treatment contrasts, with the vmPFC lesion patients as the reference group to compare each control group with, whereas recipient was coded using sum-to-zero contrasts.

The final models for each variable were:

Credits ~ Recipient * Group + (1|ID) with gamma log link function

Choice ~ Effort + Reward + Recipient + Group + Effort:Group + Reward:Recipient + Recipient:Group + Effort:Reward + (1 + Reward + Recipient + Effort + Reward:Recipient + Effort:Reward | ID) with binomial link function

K ~ Recipient * Group + (1|ID) with gamma log link function

β ~ Recipient * Group + (1|ID)

Force ~ Effort * Recipient * Reward * Group + (1 + Effort + Recipient + Reward +
Reward:Recipient | ID)

We used the parameters package[2] (v0.18.1; model_parameters function) to extract standardised model coefficients (exponentiated in GLMMs to generate odds ratios for choices and mean ratios for K parameters), their standard errors, and 95% confidence intervals. Significant interactions were explored using estimated marginal means for categorical predictors and estimated marginal trends for continuous predictors (emmeans / emmtrends; emmeans package[3] v1.7.2).

Computational model space

Based on all combinations of discount functions and whether parameters were recipient specific, the full model space was:

Model 1: Parabolic, $1K1\beta$
Model 2: Linear, $1K1\beta$
Model 3: Hyperbolic, $1K1\beta$
Model 4: Parabolic, $1K2\beta$
Model 5: Linear, $1K2\beta$
Model 6: Hyperbolic, $1K2\beta$
Model 7: Parabolic, $2K1\beta$
Model 8: Linear, $2K1\beta$
Model 9: Hyperbolic, $2K1\beta$
Model 10: Parabolic, $2K2\beta$
Model 11: Linear, $2K2\beta$
Model 12: Hyperbolic, $2K2\beta$

The linear, hyperbolic and parabolic models were specified as follows:

(a) Parabolic: $SV_{(t)} = R_{(t)} - \left(K * {E_{(t)}}^2\right)$
(b) Linear: $SV_{(t)} = R_{(t)} - \left(K * E_{(t)}\right)$
(c) Hyperbolic: $SV_{(t)} = \frac{R_{(t)}}{1 + \left(K * E_{(t)}\right)}$

The models assumed that the subjective value (SV) of the offer on trial (t) is determined by the effort level (E) (scaled to the proportion of the MVC) and reward level (R) (the number of credits) and the subject-specific discounting parameter (*K*), which describes the steepness of each individual's devaluation of rewards by effort. Thus, the higher the K value, the steeper the discount function. Note that each individual's discounting function is referenced to the SV of the baseline offer (which was always 1).

The softmax function was defined as:

$$\Pr(i) = \frac{e^{\beta . SV_i}}{e^{\beta} + e^{\beta . SV_i}}$$

where Pr(i) represents the probability of choosing option *i* that has a subjective value of (i), and β is the softmax parameter that defines the consistency (or inverse stochasticity) of each participant's choices.

Model fitting

The hierarchical MAP approach first fits the data at the individual subject level using MLE and then implements a second, higher level across the full sample. MLE at the first level provides the expectation, calculating the log-likelihood of the choices for each participant, given the model. Next, the maximum posterior probability estimate is computed using the observed choices and prior from the group-level Gaussian. Initial group-level Gaussians are uninformative priors (mean=0.1 plus noise, variance=100) then this distribution is recomputed during maximisation and the expectation and maximisation steps are iteratively repeated. We repeated these steps until the group-summed posterior likelihood converged (relative change in posterior likelihood <0.001) or the maximum number of iterations (800) was reached. To ensure accurate estimation and a plausible range of parameter values, we used appropriate transfer functions to transform estimates from Gaussian to native model space[4,5].

We identified the best fitting model (parabolic $2K2\beta$) based on complementary evidence from Bayesian model comparison, based on the integrated Bayesian Information Criterion (BICint; lower is better[6,7]) and the exceedance probability (XP; higher is better[8]). The XP represents the posterior probability that each model is the most likely of the model set in the population and over 0.95 is considered strong evidence in favour of that model. This was calculated from random-effects analysis of the Laplace approximation of the log model evidence using the spm_BMS routine[9] from SPM8 (http://www.fil.ion.ucl.ac.uk/spm/software/spm8/). We additionally used the mbb-vb-toolbox[10] (http://mbb-team.github.io/VBA-toolbox/) to estimate the expected model frequency (EF) across all participants and the XP and EF in each group separately. This was part of a between-groups random effects analysis that also formally compared EF between pairs of groups and provided the probability that the same model is best in both groups. Finally, we calculated R^2 for each model as the squared median choice probability across trials and participants.

Table S1. *Summary of demographic variables for each group and between-group comparisons*

**Variable**	**HC** mean [SD]	**vmPFC** mean [SD]	**LC** mean [SD]	**HC - vmPFC**	**LC - vmPFC**	**HC - LC**
Age	60.00 [8.11]	56.44 [10.95]	56.00 [11.81]	0.19	0.99	0.20
Gender	42% M	44% M	33% M	0.91	0.52	0.55
Edu	4.08 [1.14]	2.84 [1.11]	3.27 [1.33]	<0.001	0.40	0.03
AMI	1.19 [0.50]	1.41 [0.57]	1.32 [0.60]	0.14	0.99	0.30
BDI	7.75 [9.34]	10.52 [8.27]	14.27 [11.54]	0.11	0.36	0.04
Trail A	23.37 [8.16]	26.28 [9.50]	29.24 [13.64]	0.20	0.65	0.16
Trail B	57.17 [29.76]	66.90 [41.15]	75.32 [37.25]	0.40	0.37	0.08

Note. HC: Healthy controls, LC: Lesion controls, SD: standard deviation of the mean, Edu: Education scale (1-6); AMI: Apathy Motivation Index, BDI: Beck Depression Inventory; Trial A / B: Trail Making Test Part / Part B, M: male; *p* values for comparisons between groups from Wilcoxon two-sided signed rank tests

Table S2. *Generalised linear mixed-effects model predicting credits*

Parameter	b	SE	CI low	CI high	*t*	*p*	LC n=20	Edu
(Intercept)	299.50	21.23	260.36	344.53	80.44	<0.001	<0.001	<0.001
Recipient (Self vs. Other)	1.25	0.04	1.17	1.33	6.59	<0.001	<0.001	<0.001
Group (vmPFC vs. HC)	1.20	0.11	1.01	1.44	2.03	0.044	0.10	0.12
Group (vmPFC vs. LC)	1.15	0.13	0.91	1.44	1.19	0.23	0.77	0.27
Recipient * Group (vmPFC vs. HC)	0.88	0.04	0.81	0.95	-3.07	0.003	0.001	0.003
Recipient * Group (vmPFC vs. LC)	0.87	0.05	0.78	0.97	-2.60	0.010	<0.001	0.011

Note. HC: Healthy controls, LC: Lesion controls. LC n=20: supplementary analysis including the five additional patients with damage on the medial wall, but not focal to vmPFC, in the lesion control group (n=20). Results did not change compared to without these patients. Edu: supplementary analysis controlling for participants' levels of education by including this as a fixed effect in the model (main effect of education on credits p=0.47).

Table S3. *Generalised linear mixed-effects model predicting choices*

Parameter	OR	SE	CI low	CI high	*z*	*p*	LC n=20	Edu
(Intercept)	14.25	6.10	6.16	32.95	6.21	<0.001	<0.001	<0.001
Effort	0.40	0.08	0.27	0.60	-4.43	<0.001	<0.001	<0.001
Reward	6.91	1.36	4.70	10.15	9.83	<0.001	<0.001	<0.001
Recipient (Self vs. Other)	5.73	1.22	3.77	8.70	8.18	<0.001	<0.001	<0.001
Group (vmPFC vs. HC)	1.83	0.90	0.70	4.82	1.23	0.22	0.21	0.16
Group (vmPFC vs. LC)	2.09	1.32	0.60	7.21	1.16	0.25	0.35	0.23
Effort * Group (vmPFC vs. HC)	0.44	0.10	0.28	0.69	-3.57	<0.001	<0.001	<0.001
Effort * Group (vmPFC vs. LC)	0.73	0.21	0.42	1.27	-1.11	0.27	0.40	0.26
Reward * Recipient (Self vs. Other)	1.71	0.14	1.46	2.01	6.56	<0.001	<0.001	<0.001
Recipient * Group (vmPFC vs. HC)	0.60	0.14	0.38	0.94	-2.22	0.026	0.022	0.044
Recipient * Group (vmPFC vs. LC)	0.48	0.14	0.27	0.84	-2.57	0.010	0.003	0.010
Effort * Reward	0.93	0.06	0.82	1.06	-1.06	0.29	0.27	0.35

Note. HC: Healthy controls, LC: Lesion controls. LC n=20: supplementary analysis including the five additional patients with damage on the medial wall, but not focal to vmPFC, in the lesion control group (n=20). Results did not change compared to without these patients. Edu: supplementary analysis controlling for participants' levels of education by including this as a fixed effect in the model (main effect of education on choices p=0.51).

Table S4. *Generalised linear mixed-effects model predicting K parameters*

Parameter	b	SE	CI low	CI high	*t*	*p*	LC n=20	Edu
(Intercept)	0.08	0.02	0.05	0.12	-13.18	<0.001	<0.001	<0.001
Recipient (Self vs. Other)	0.54	0.04	0.46	0.64	-7.71	<0.001	<0.001	<0.001
Group (vmPFC vs. HC)	0.84	0.21	0.52	1.36	-0.72	0.47	0.48	0.35
Group (vmPFC vs. LC)	0.67	0.21	0.36	1.24	-1.28	0.20	0.30	0.17
Recipient * Group (vmPFC vs. HC)	1.36	0.13	1.12	1.65	3.17	0.002	0.002	0.002
Recipient * Group (vmPFC vs. LC)	1.46	0.18	1.15	1.85	3.13	0.002	<0.001	0.002

Note. HC: Healthy controls, LC: Lesion controls. LC n=20: supplementary analysis including the five additional patients with damage on the medial wall, but not focal to vmPFC, in the lesion control group (n=20). Results did not change compared to without these patients. Edu: supplementary analysis controlling for participants' levels of education by including this as a fixed effect in the model (main effect of education on *K* parameters *p*=0.50).

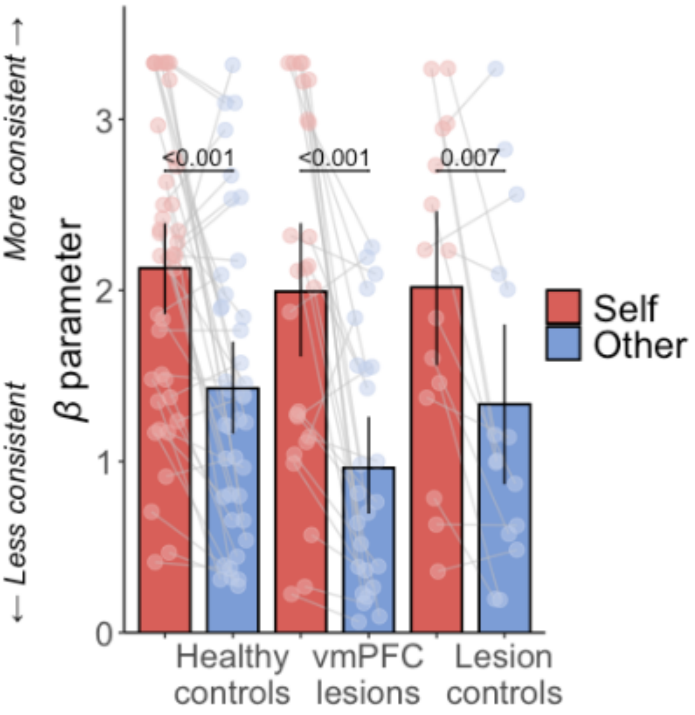

Figure S1. Choice consistency is higher for self than other but no significant effect of vmPFC damage. Analysis of choice consistency β parameters revealed choices in all three groups more consistently followed the relative subjective value between options. However, there were no significant differences between groups in choice consistency overall and no significant interactions between group and recipient in predicting β parameters (see Table S5). Data are presented as mean values +/- SEM. Dots show individual data points for each participant (n=80). Significance lines between self and other represent differences within each group in post-hoc comparisons (two-sided, *p*s<0.007 uncorrected: follow-ups of significant GLMM interactions).

Table S5. *Generalised linear mixed-effects model predicting β parameters*

Parameter	b	SE	CI low	CI high	*t*	*p*	LC n=20	Edu
(Intercept)	0.83	0.13	0.61	1.12	-1.24	0.22	0.27	0.57
Recipient (Self vs. Other)	1.69	0.16	1.40	2.05	5.41	<0.001	<0.001	<0.001
Group (vmPFC vs. HC)	1.36	0.27	0.92	2.01	1.56	0.12	0.12	0.54
Group (vmPFC vs. LC)	1.22	0.31	0.74	2.02	0.80	0.42	0.59	0.57
Recipient * Group (vmPFC vs. HC)	0.85	0.10	0.66	1.08	-1.35	0.18	0.18	0.18
Recipient * Group (vmPFC vs. LC)	0.84	0.13	0.61	1.15	-1.12	0.27	0.056	0.27

Note. HC: Healthy controls, LC: Lesion controls. LC n=20: supplementary analysis including the five additional patients with damage on the medial wall, but not focal to vmPFC, in the lesion control group (n=20). Results did not change compared to without these patients. Edu: supplementary analysis controlling for participants' levels of education by including this as a fixed effect in the model (main effect of education on *β* parameters p=0.057).

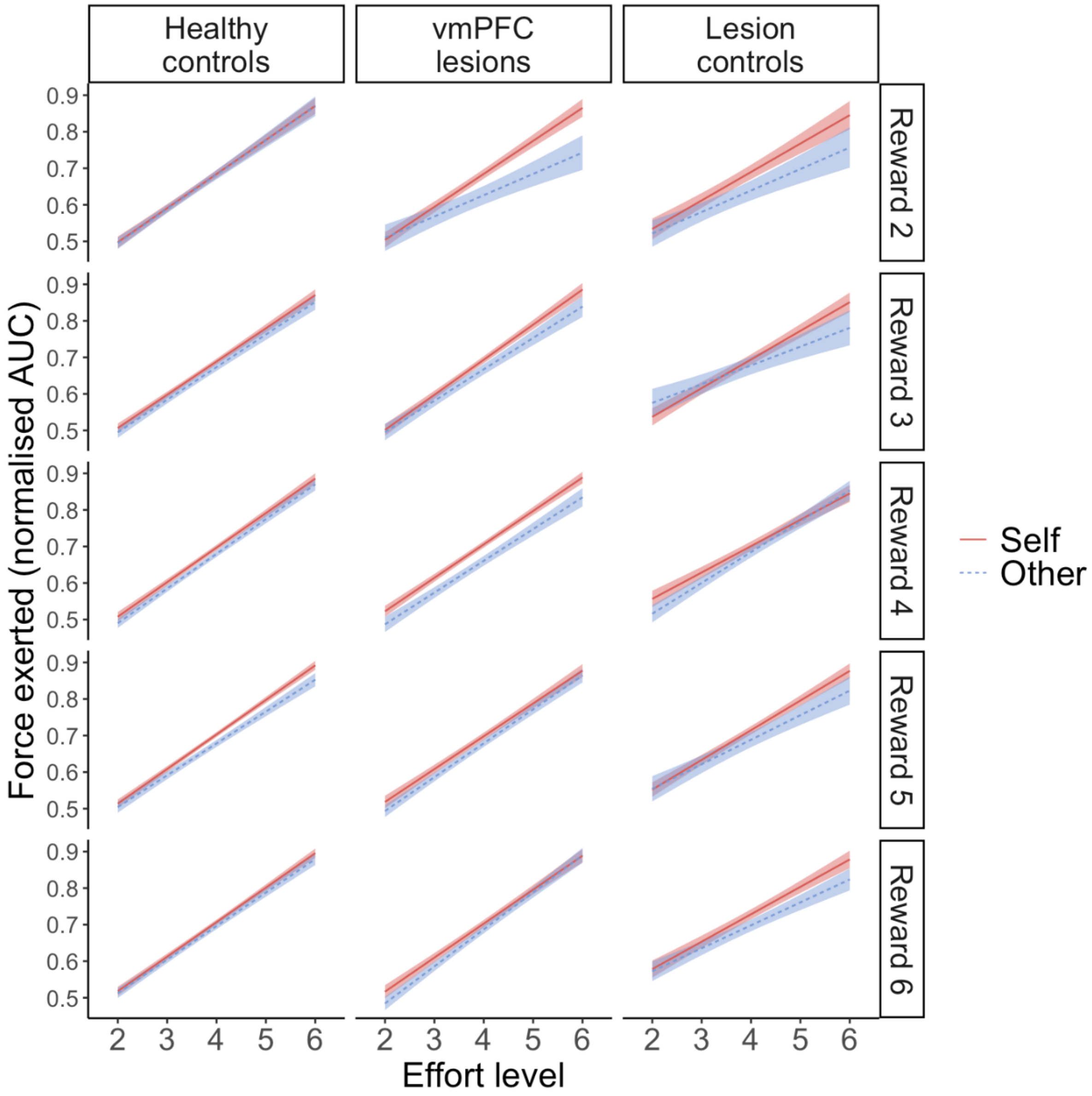

Figure S2. vmPFC damage decreases the force exerted to gain rewards for another person, compared to oneself, as the effort required increases, particularly for the smallest rewards. In addition to the group*recipient and group*recipient*effort interactions shown in Figure 5, the linear mixed-effects model of force exerted showed a significant 4-way group*recipient*effort*reward interaction for vmPFC patients compared to healthy controls ($b_{(8638)}$=0.05 [0.03, 0.08], p<0.001; Table S5). The corresponding interaction for vmPFC patients compared to lesion patients was not significant ($b_{(8638)}$=0.02 [-0.01, 0.05], p=0.17). This sensitivity to the reward available and effort required, combined with high overall success rates, suggests vmPFC patients' reduced willingness to exert force for prosocial rewards was not due to an inability to meet the required force or attend to the information in the trial. Data are presented as GLM estimated conditional means +/- 95% confidence interval.

Table S6. *Linear mixed-effects model predicting force*

Parameter	b	SE	CI low	CI high	*t*	*p*	LC n=20	Edu
(Intercept)	-0.08	0.07	-0.21	0.05	-1.19	0.23	0.36	0.067
Effort	0.78	0.04	0.70	0.86	18.26	<0.001	<0.001	<0.001
Recipient (Self vs. Other)	0.13	0.03	0.06	0.20	3.62	<0.001	<0.001	<0.001
Reward	0.04	0.01	0.01	0.06	3.16	0.002	0.002	0.002
Group (vmPFC vs. HC)	0.10	0.09	-0.07	0.27	1.20	0.23	0.25	0.047
Group (vmPFC vs. LC)	0.12	0.11	-0.10	0.33	1.05	0.29	0.93	0.19
Effort * Group (vmPFC vs. HC)	0.07	0.05	-0.04	0.17	1.21	0.23	0.23	0.24
Effort * Group (vmPFC vs. LC)	-0.13	0.07	-0.27	0.00	-1.94	0.053	0.088	0.054
Effort * Recipient (Self vs. Other)	0.03	0.01	0.01	0.05	2.89	0.004	0.004	0.004
Recipient * Group (vmPFC vs. HC)	-0.09	0.04	-0.18	-0.01	-2.10	0.036	0.032	0.036
Recipient * Group (vmPFC vs. LC)	-0.05	0.06	-0.16	0.05	-0.97	0.33	0.22	0.32
Reward * Group (vmPFC vs. HC)	0.00	0.01	-0.03	0.03	-0.18	0.86	0.84	0.87
Reward * Group (vmPFC vs. LC)	0.03	0.02	0.00	0.07	1.73	0.084	0.066	0.082
Effort * Reward	0.01	0.01	-0.01	0.03	1.03	0.30	0.30	0.31
Recipient * Reward	-0.01	0.01	-0.03	0.02	-0.53	0.59	0.54	0.61
Effort * Reward * Group (vmPFC vs. HC)	-0.03	0.01	-0.05	0.00	-2.05	0.041	0.042	0.039
Effort * Reward * Group (vmPFC vs. LC)	-0.02	0.02	-0.05	0.01	-1.51	0.13	0.18	0.14
Effort * Recipient * Group (vmPFC vs. HC)	-0.03	0.01	-0.05	0.00	-2.09	0.037	0.041	0.042
Effort * Recipient * Group (vmPFC vs. LC)	0.00	0.02	-0.03	0.03	0.24	0.81	0.83	0.81
Effort * Recipient * Reward	-0.04	0.01	-0.05	-0.02	-3.57	<0.001	<0.001	<0.001
Recipient * Reward * Group (vmPFC vs. HC)	0.02	0.02	-0.01	0.05	1.55	0.12	0.11	0.12
Recipient * Reward * Group (vmPFC vs. LC)	0.01	0.02	-0.03	0.04	0.34	0.73	0.66	0.75
Effort * Recipient * Reward * Group (vmPFC vs. HC)	0.05	0.01	0.03	0.08	4.21	<0.001	<0.001	<0.001
Effort * Recipient * Reward * Group (vmPFC vs. LC)	0.02	0.02	-0.01	0.05	1.38	0.17	0.28	0.18

Note. HC: Healthy controls, LC: Lesion controls. LC n=20: supplementary analysis including the five additional patients with damage on the medial wall, but not focal to vmPFC, in the lesion control group (n=20). Results did not change compared to without these patients. Edu: supplementary analysis controlling for participants' levels of education by including this as a fixed effect in the model (main effect of education on force p=0.020).

Table S7. *Linear mixed-effects model predicting success*

**Parameter**	**b**	**SE**	**CI low**	**CI high**	***t***	***p***	**LC n=20**	**Edu**
(Intercept)	-0.30	0.16	-0.62	0.02	-1.86	0.064	0.32	0.20
Recipient (Self vs. Other)	0.35	0.11	0.13	0.57	3.17	0.002	0.001	0.002
Group (vmPFC vs. HC)	0.54	0.21	0.14	0.95	2.63	0.009	0.059	0.085
Group (vmPFC vs. LC)	0.12	0.26	-0.40	0.64	0.46	0.65	0.63	0.79
Recipient * Group (vmPFC vs. HC)	-0.32	0.14	-0.59	-0.04	-2.30	0.023	0.020	0.026
Recipient * Group (vmPFC vs. LC)	-0.18	0.18	-0.53	0.17	-1.03	0.30	0.13	0.31

Note. HC: Healthy controls, LC: Lesion controls. LC n=20: supplementary analysis including the five additional patients with damage on the medial wall, but not focal to vmPFC, in the lesion control group (n=20). Results did not change compared to without these patients. Edu: supplementary analysis controlling for participants' levels of education by including this as a fixed effect in the model (main effect of education on success p=0.17).

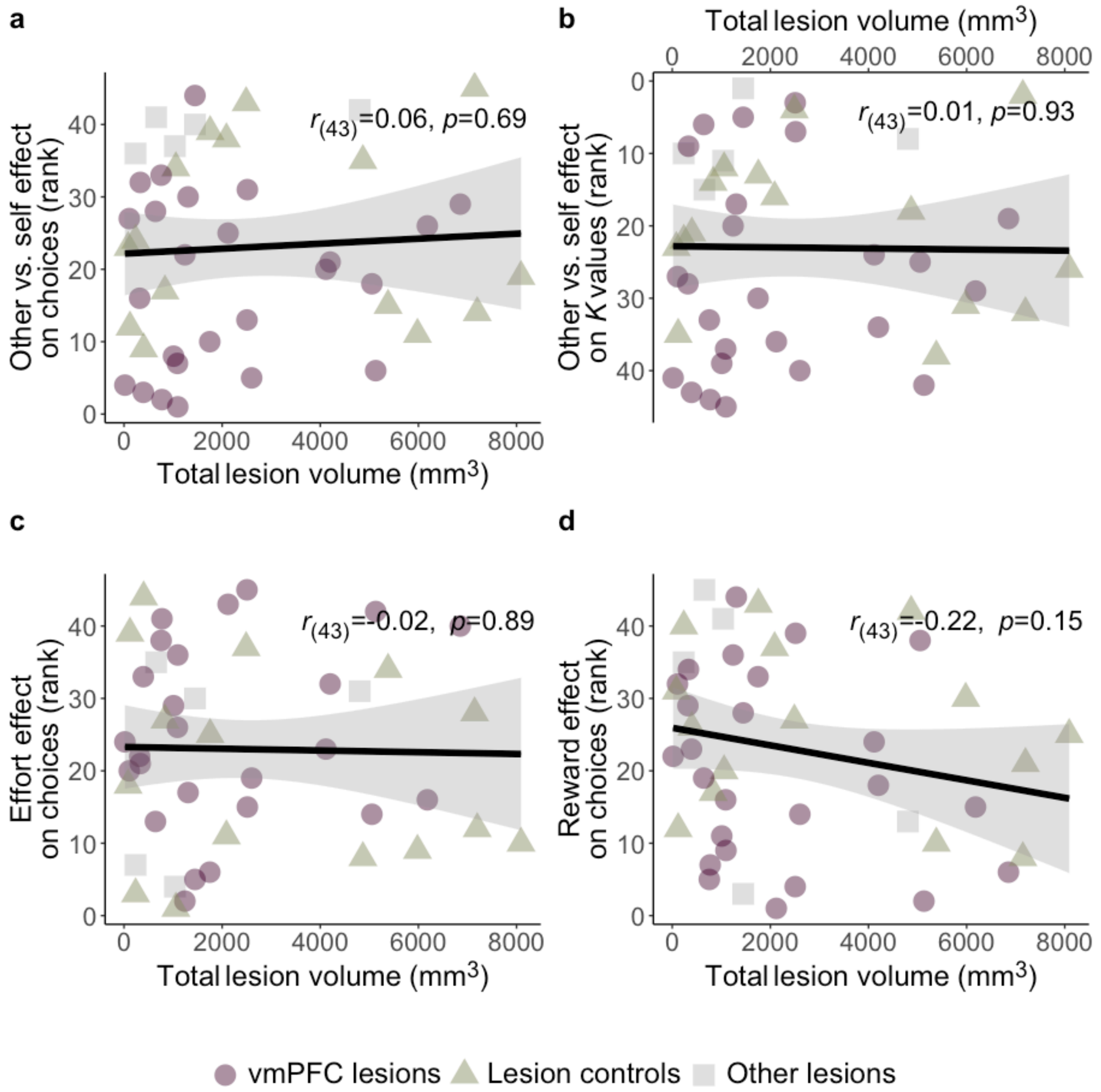

Figure S3. No significant correlations between total lesion size and the behavioural predictors for VLSM. As a control analysis to support the voxel-based lesion symptom mapping (VLSM), we ran Pearson correlations between total lesion volume and each of the four behavioural predictors, calculated as the (ranked) participant-level random effects from the generalised linear mixed-effects models of choices and *K* parameters (effect of recipient on choices, effect of reward on choices, effect of effort on choices, effect of recipient on *K* parameters). The lack of significant correlations (uncorrected *p*s>0.15) suggests that overall lesion size did not confound the VLSM analysis and results are specific to damage in each of the identified subregions. Lines show estimated conditional means +/- 95% confidence interval.

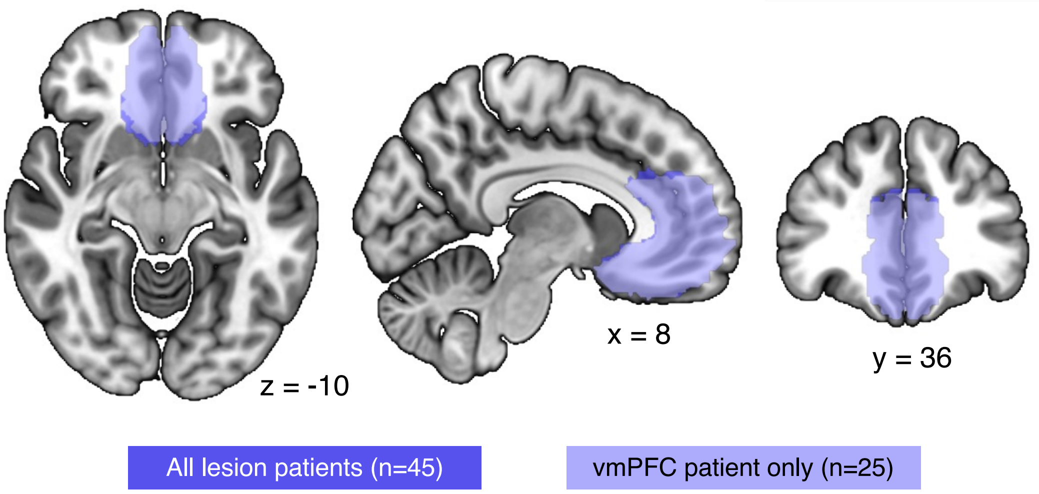

Figure S4. Voxels included in voxel-based lesion-symptom mapping (VLSM) analysis where at least five patients had damage. We conducted VLSM once in all patients with damage (n=45) shown in darker purple and once limited to patients in the vmPFC group (n=20; see Methods).

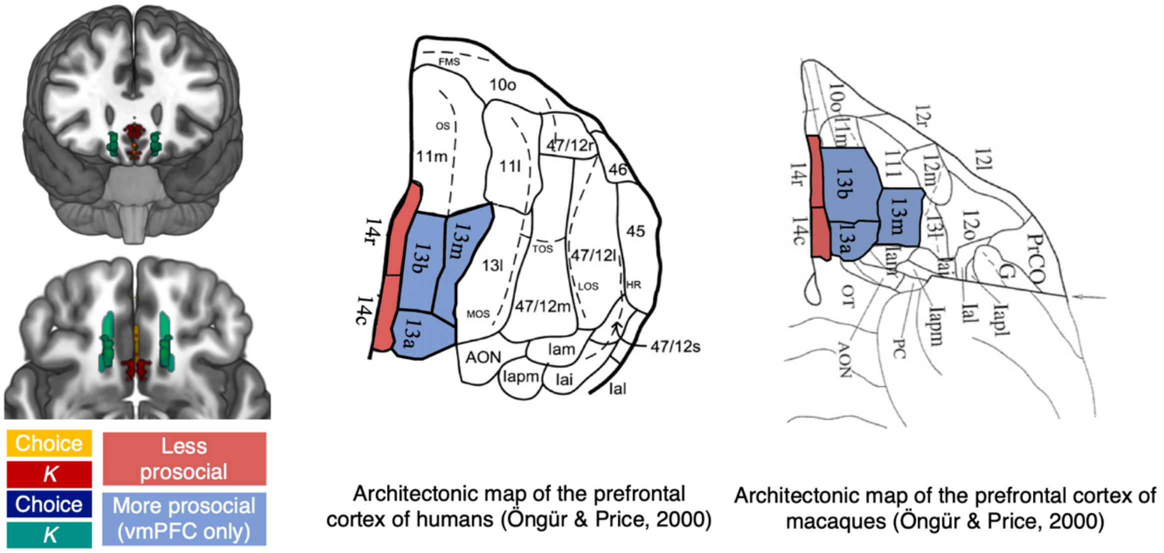

Figure S5. Architectonic maps of the prefrontal cortex in humans and macaques showing divisions between area 14 (or 14m which comprises 14r and 14c) and area 13 (comprising 13a, 13b and 13m). Voxel-based lesion mapping showed two adjacent but distinct areas of vmPFC where motivation in patients with damage was either less prosocial (red / yellow) or relatively more prosocial (blue / green; in vmPFC patients only analysis). These areas correspond to separate cytoarchitectonic regions: area 14 on the medial wall (alternatively labelled 14m and comprising 14r and 14c; red) and area 13 on the orbital gyrus (comprising 13a, 13b and 13m, blue). These anatomical divisions are preserved across species[11,12]. Adapted with permission from ref. 11, Oxford University Press.

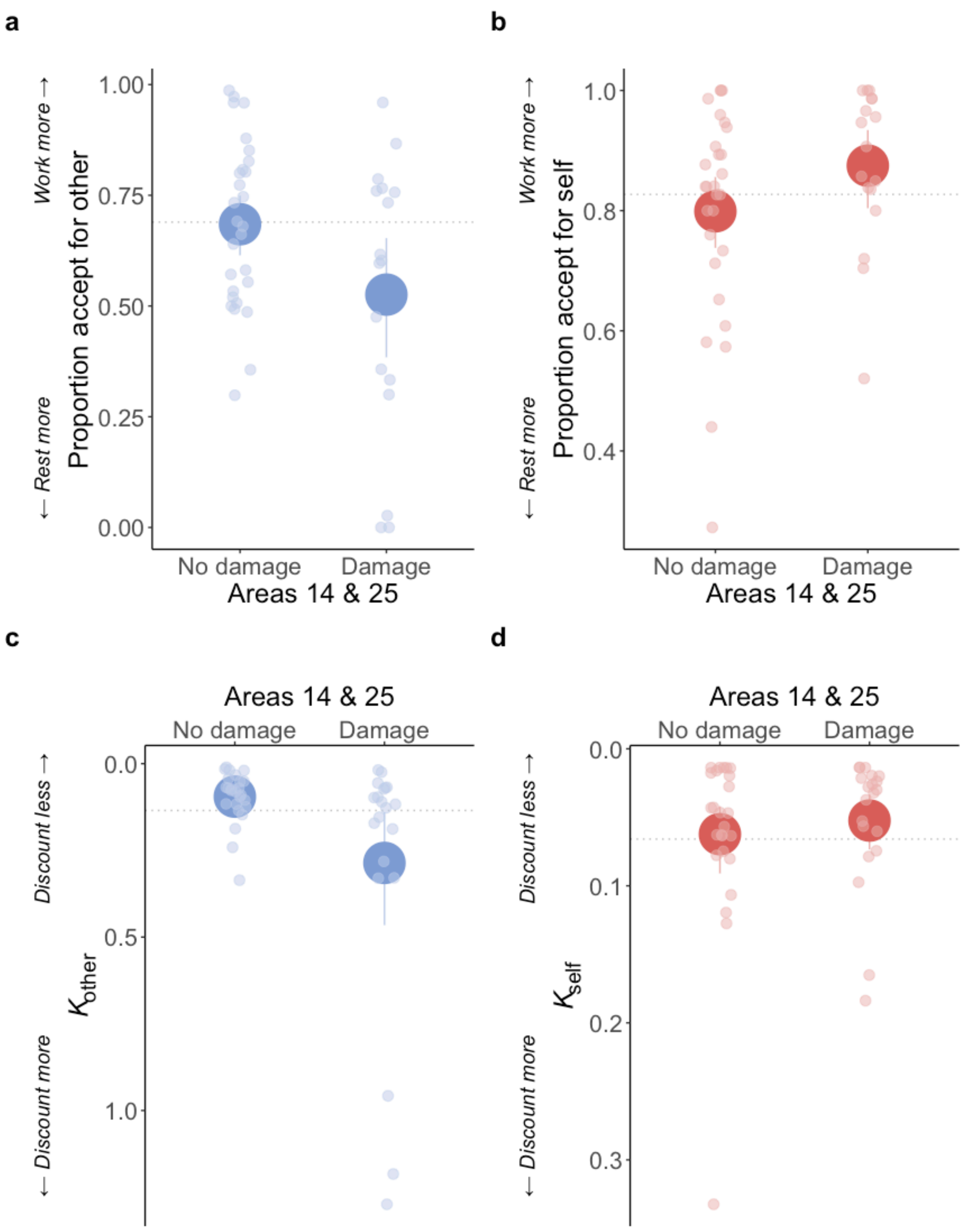

Figure S6. Damage to medial vmPFC regions decreases willingness to exert effort for others and increases willingness to obtain self-benefitting rewards. To further interpret the voxel-based lesion symptom mapping (VLSM) finding of decreased prosociality with damage to portions of vmPFC on the medial wall (areas 14 and 25), we plotted each recipient separately to examine how damage affects **(a)** choices for other **(b)** choices for self **(c)** discounting *K* parameters for other and **(d)** discounting *K* parameters for self (n=40 participants). We categorised participants by whether they had damage in the areas identified in the VLSM analysis for each effect (choices and *K*; see Figure 6A). Patients with no damage to the identified region had damage elsewhere in the brain. These plots suggest that decreased prosociality shown by the recipient effects (other vs. self) in the VLSM are driven by both lower willingness to help the other person and higher willingness to work for oneself in patients with damage to these regions. Data are presented as mean values +/- SEM. The dotted line represents the mean value for the healthy control group for comparison.

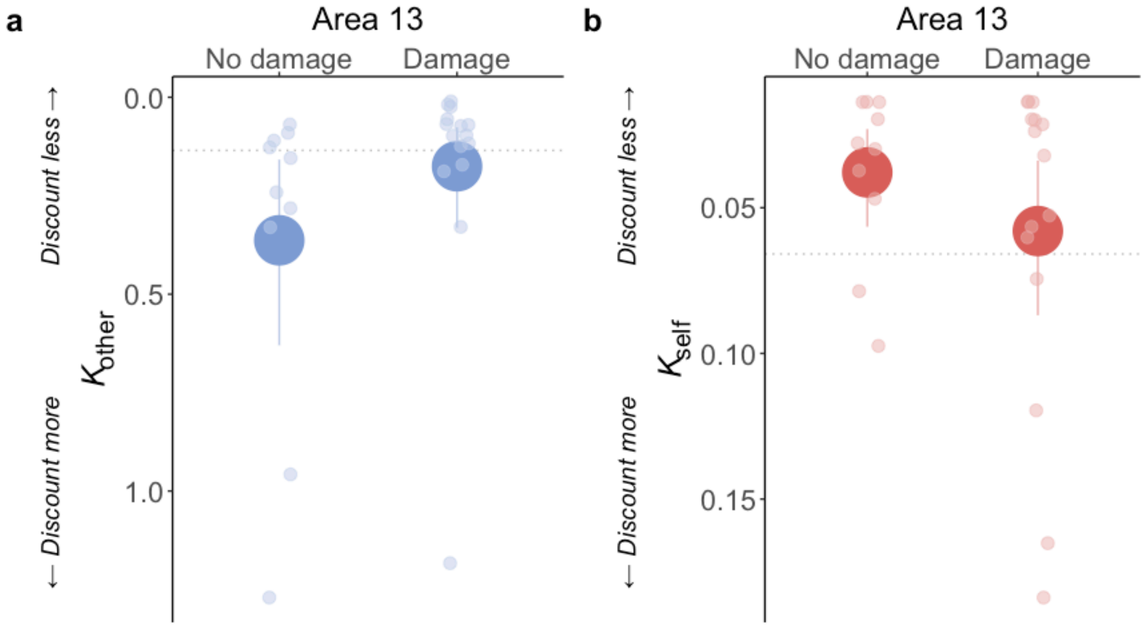

Figure S7. Damage to lateral vmPFC compared to other vmPFC subregions leads to relative increases in willingness to help others. Our voxel-based lesion symptom mapping (VLSM) analysis identified a lateral portion of vmPFC in area 13 where damage was, in contrast, associated with relatively increased prosociality. To further interpret this finding, we extracted the extent of damage for each participant in the region identified in the VLSM analysis of the recipient effect (other vs. self) on *K* parameters (see Figure 7A). The corresponding effect on choices was associated with a smaller, overlapping region of area 13. Plotting damage in this region against **(a)** discounting *K* parameter for other and **(b)** discounting *K* parameter for self separately shows the relative increase in prosociality is driven by both lower discounting (higher willingness to work) for other and higher discounting (lower willingness to work) to some extent for self (n=25 participants). Data are presented as mean values +/- SEM. The dotted line represents the mean value for the healthy control group for comparison.

References

1. Voeten, C. *buildmer: Stepwise Elimination and Term Reordering for Mixed-Effects Regression*. (2022).
2. Lüdecke, D., Ben-Shachar, M. S., Patil, I. & Makowski, D. Extracting, Computing and Exploring the Parameters of Statistical Models using R. *Journal of Open Source Software* **5**, 2445 (2020).
3. Lenth, R. emmeans: Estimated Marginal Means, aka Least-Squares Means. (2021).
4. Daw, N. D. Trial-by-trial data analysis using computational models. in *Decision Making, Affect, and Learning: Attention and Performance XXIII* (eds. Delgado, M. R., Phelps, E. A. & Robbins, T. W.) (OUP Oxford, 2009).
5. Lockwood, P. L. & Klein-Flügge, M. C. Computational modelling of social cognition and behaviour—a reinforcement learning primer. *Soc Cogn Affect Neurosci* (2020) doi:10.1093/scan/nsaa040.
6. Wittmann, M. K. *et al.* Global reward state affects learning and activity in raphe nucleus and anterior insula in monkeys. *Nature Communications* **11**, 3771 (2020).
7. Huys, Q. J. M. *et al.* Disentangling the Roles of Approach, Activation and Valence in Instrumental and Pavlovian Responding. *PLOS Computational Biology* **7**, e1002028 (2011).
8. MacKay, D. J. C. *Information Theory, Inference and Learning Algorithms*. (Cambridge University Press, 2003).
9. Stephan, K. E., Penny, W. D., Daunizeau, J., Moran, R. J. & Friston, K. J. Bayesian Model Selection for Group Studies. *Neuroimage* **46**, 1004–1017 (2009).
10. Daunizeau, J., Adam, V. & Rigoux, L. VBA: A Probabilistic Treatment of Nonlinear Models for Neurobiological and Behavioural Data. *PLOS Computational Biology* **10**, e1003441 (2014).
11. Öngür, D. & Price, J. L. The Organization of Networks within the Orbital and Medial Prefrontal Cortex of Rats, Monkeys and Humans. *Cerebral Cortex* **10**, 206–219 (2000).
12. Carmichael, S. T. & Price, J. L. Architectonic subdivision of the orbital and medial prefrontal cortex in the macaque monkey. *J Comp Neurol* **346**, 366–402 (1994).
