# Supplementary material for: Human ventromedial prefrontal cortex is necessary for prosocial motivation
Source: Nat Hum Behav. 2024 May 27;8(7):1403–16. doi: 10.1038/s41562-024-01899-4 (PMC11272586; doi:10.1038/s41562-024-01899-4)
Supplement: Supplementary file 2 — Reporting Summary [file 41562_2024_1899_MOESM2_ESM.pdf]

nature portfolio

Corresponding author(s): Dr Patricia Lockwood & Dr Jo Cutler

Last updated by author(s): Apr 8, 2024

Reporting Summary

Nature Portfolio wishes to improve the reproducibility of the work that we publish. This form provides structure for consistency and transparency in reporting. For further information on Nature Portfolio policies, see our Editorial Policies and the Editorial Policy Checklist.

Statistics

For all statistical analyses, confirm that the following items are present in the figure legend, table legend, main text, or Methods section.

n/a	Confirmed	
☐	☒	The exact sample size (*n*) for each experimental group/condition, given as a discrete number and unit of measurement
☐	☒	A statement on whether measurements were taken from distinct samples or whether the same sample was measured repeatedly
☐	☒	The statistical test(s) used AND whether they are one- or two-sided<br>*Only common tests should be described solely by name; describe more complex techniques in the Methods section.*
☐	☒	A description of all covariates tested
☐	☒	A description of any assumptions or corrections, such as tests of normality and adjustment for multiple comparisons
☐	☒	A full description of the statistical parameters including central tendency (e.g. means) or other basic estimates (e.g. regression coefficient) AND variation (e.g. standard deviation) or associated estimates of uncertainty (e.g. confidence intervals)
☐	☒	For null hypothesis testing, the test statistic (e.g. *F*, *t*, *r*) with confidence intervals, effect sizes, degrees of freedom and *P* value noted<br>*Give P values as exact values whenever suitable.*
☐	☒	For Bayesian analysis, information on the choice of priors and Markov chain Monte Carlo settings
☐	☒	For hierarchical and complex designs, identification of the appropriate level for tests and full reporting of outcomes
☒	☐	Estimates of effect sizes (e.g. Cohen's *d*, Pearson's *r*), indicating how they were calculated

Our web collection on statistics for biologists contains articles on many of the points above.

Software and code

Policy information about availability of computer code

Data collection	Psychtoolbox version 3 and Qualtrics (website, no versions)
Data analysis	MATLAB 2019b, R (version 3.6.2) with R studio (version 1.4.1106) and FSL (version 6.0.6.2).<br><br>Code availability<br><br>Code for modelling and analysis is available at https://doi.org/10.17605/osf.io/xdnek

For manuscripts utilizing custom algorithms or software that are central to the research but not yet described in published literature, software must be made available to editors and reviewers. We strongly encourage code deposition in a community repository (e.g. GitHub). See the Nature Portfolio guidelines for submitting code & software for further information.

Data

Policy information about availability of data

All manuscripts must include a data availability statement. This statement should provide the following information, where applicable:

- Accession codes, unique identifiers, or web links for publicly available datasets
- A description of any restrictions on data availability
- For clinical datasets or third party data, please ensure that the statement adheres to our policy

Data are available at https://doi.org/10.17605/osf.io/xdnek

Research involving human participants, their data, or biological material

Policy information about studies with human participants or human data. See also policy information about sex, gender (identity/presentation), and sexual orientation and race, ethnicity and racism.

Reporting on sex and gender	The three groups were matched on self-reported gender: 25 patients with vmPFC damage (mean age=56.44; 14 females), 15 lesion control (LC) patients with damage to areas outside vmPFC (mean age=56.00; 10 females), and 40 healthy control (HC) participants (mean age=60.00; 23 females). No further analysis on sex or gender was required for the research questions and as splitting the samples based on any characteristic would compromise statistical power.
Reporting on race, ethnicity, or other socially relevant groupings	These variables were not included as they were not required for the research and as splitting the samples based on any characteristic would compromise statistical power.
Population characteristics	Please see behavioural & social sciences study design section
Recruitment	We recruited three groups of participants, one with focal damage to vmPFC and one with lesions elsewhere, from a database of 453 neurological patients, as well as healthy age and gender-matched controls from university databases and the community. Self-selection bias of bias due to the requirement of ability to attend in-person experiment using physical effort mean the sample only includes participants with less severe effects of brain lesions on motivation, a more conservative test of group differences than if vmPFC patients with most severe impacts tested.
Ethics oversight	The research complied with all relevant ethical regulations and the Oxford University Medical Sciences Inter Divisional Research Ethics Committee and National Health Service Health Research Authority approved the protocol (Ethics Ref. 18/LO/2152).

Note that full information on the approval of the study protocol must also be provided in the manuscript.

Field-specific reporting

Please select the one below that is the best fit for your research. If you are not sure, read the appropriate sections before making your selection.

☐ Life sciences ☒ Behavioural & social sciences ☐ Ecological, evolutionary & environmental sciences

For a reference copy of the document with all sections, see nature.com/documents/nr-reporting-summary-flat.pdf

Behavioural & social sciences study design

All studies must disclose on these points even when the disclosure is negative.

Study description	Quantitative experimental with naturally occurring groups (see below). Participants in each of three groups completed a effort-based decision-making task that manipulated effort, reward and recipient (self or other) independently. Dependent variables measured were the number of credits earned, choices to exert effort, force exerted and computational parameters from effort discounting models.
Research sample	We compared 25 patients with vmPFC damage (mean age=56.44; 14 females) to two control groups. 15 lesion control (LC) patients had damage to areas outside vmPFC (mean age=56.00; 10 females), and 40 healthy control (HC) participants did not have any brain damage (mean age=60.00; 23 females). Patients across both lesion groups were recruited from a database of 453 neurological patients with lesion control patients and healthy controls selected to age and gender-match the vmPFC patient group. The sample was not recruited to be representative of any specific population but the rationale was to enable us to test the impact of vmPFC lesions specifically, compared to patients with lesions elsewhere and participants without brain damage.
Sampling strategy	Convenience sample from neurological database based on lesion location (vmPFC or lesion control) and ability to complete an in-person study using an effort-based decision making task. No sample size calculation was performed. The sample sizes were chosen based on the maximum number of vmPFC and lesion control patients possible, as these patients are extremely rare and a difficult sample to recruit and test. The sample sizes are approximately double the number used in most previous research and the research is also sufficiently powered given the substantial number of trials in the experimental task.

Data collection	Data were collected on a computer. Participants attended a single in-person testing session that started with an assessment with a neurologist (SGM). Experimenters were therefore not blind to group.
Timing	March 2017 - August 2019
Data exclusions	Two other patients with vmPFC lesions took part but were excluded from all analyses for not following the task instructions or not understanding the task. Five additional patients with lesions affecting more dorsal regions of mPFC or the anterior cingulate cortex (ACC) also completed the task (age range=49-66, mean=57.40; 2 females). These patients were only included in the lesion mapping analysis (see below), which had a total sample of 45 patients. We did not include patients with dorsal mPFC or ACC lesions in the main between-group analyses, as the damage was not within the vmPFC region of interest, but also was not distinct enough to include in the lesion control group.
Non-participation	No participants dropped out.
Randomization	Allocation to groups based on lesion location (vmPFC or lesion control) or no lesion (healthy controls). The three groups were carefully matched, with no differences in gender, age, cognitive ability, or levels of apathy. The two lesion groups also did not differ from each other in education or depression (see Methods and Table S1). However, the vmPFC and LC groups did differ from healthy controls in level of education. We therefore repeated all our behavioural analysis controlling for education as a covariate. Controlling for education did not change any of our key results or inferences regarding group differences in prosocial behaviour (see Tables S2-S7).

Reporting for specific materials, systems and methods

We require information from authors about some types of materials, experimental systems and methods used in many studies. Here, indicate whether each material, system or method listed is relevant to your study. If you are not sure if a list item applies to your research, read the appropriate section before selecting a response.

Materials & experimental systems

n/a	Involved in the study
☒	☐ Antibodies
☒	☐ Eukaryotic cell lines
☒	☐ Palaeontology and archaeology
☒	☐ Animals and other organisms
☒	☐ Clinical data
☒	☐ Dual use research of concern
☒	☐ Plants

Methods

n/a	Involved in the study
☒	☐ ChIP-seq
☒	☐ Flow cytometry
☒	☐ MRI-based neuroimaging

Plants

Seed stocks	*Report on the source of all seed stocks or other plant material used. If applicable, state the seed stock centre and catalogue number. If plant specimens were collected from the field, describe the collection location, date and sampling procedures.*
Novel plant genotypes	*Describe the methods by which all novel plant genotypes were produced. This includes those generated by transgenic approaches, gene editing, chemical/radiation-based mutagenesis and hybridization. For transgenic lines, describe the transformation method, the number of independent lines analyzed and the generation upon which experiments were performed. For gene-edited lines, describe the editor used, the endogenous sequence targeted for editing, the targeting guide RNA sequence (if applicable) and how the editor was applied.*
Authentication	*Describe any authentication procedures for each seed stock used or novel genotype generated. Describe any experiments used to assess the effect of a mutation and, where applicable, how potential secondary effects (e.g. second site T-DNA insertions, mosiacism, off-target gene editing) were examined.*
